# Myths and methodologies: Exposure and measurements under normobaric hypoxia

**DOI:** 10.1113/EP093243

**Published:** 2026-04-08

**Authors:** Danilo Bondi

**Affiliations:** ^1^ Department of Neurosciences, Imaging and Clinical Sciences University ‘G. d'Annunzio’ Chieti–Pescara Chieti Italy

**Keywords:** carbon dioxide, continuous monitoring, flight, normobaric hypoxia, physiological fluctuation, spot value

## Abstract

Usually, settings of normobaric hypoxia underestimate the environmental hypoxia they are supposed to reproduce. The likely steady state of water vapour pressure within the human airways keeps the partial pressure of inspired oxygen very close to the assumed value, but this does not apply to the calculation of environmental hypoxia or experimental settings with other species. Additionally, in confined spaces, the CO_2_ can exceed permissible exposure limits unless sufficient ventilation is calculated and required. The conversion of parts per million (ppm) to milligrams per metre cubed in dry air is facilitated by the general formula: $\mathrm{Concentration}\;\left(\mathrm{mg}\cdot m^{-3}\right)\approx \mathrm{ppmv}\cdot M_{m}\cdot P\left(\mathrm{kPa}\right)/8314\cdot T(K)$, thus overcoming the common assumptions of dry air at 1 atm and 25°C. The use of spot values (i.e., single measurements taken at a specific point in time), especially those of oxyhaemoglobin saturation, should be avoided, given the significance of physiological fluctuations. By considering the assumptions of simulated altitude hypoxia, the calculations or estimations of the amount of carbon dioxide in the inspired air in confined spaces, and the avoidance of spot values, the physiological response will be more valid and informative.

## Abstract (highlights)

- **What is the central question of this study?**
  What are the biases that affect normobaric hypoxia studies?
- **What is the main finding and its importance?**
  Although current estimates are acceptable for inhaled oxygen, they do not accurately account for environmental oxygen. Carbon dioxide overexposure is possible, but its levels are not necessarily monitored. The practices for taking spot measurements or averaging data from continuous monitoring do not account for physiological fluctuations or the variability inherent in continuous monitoring.

## 1 INTRODUCTION

Normobaric hypoxia has been widely implemented in clinical and research settings because normobaric conditions are less expensive and more feasible than the hypobaric counterparts, either natural or simulated. Consequently, a huge debate has arisen about the difference between normobaric and hypobaric hypoxia (e.g., Debevec & Millet, 2014; Richard et al., 2014). The statement that hypobaric hypoxia induces relatively greater physiological responses than normobaric hypoxia (Millet et al., 2012) is contrasted by those arguing that physiological responses to normobaric hypoxia and hypobaric hypoxia are equivalent (Mounier & Brugniaux, 2012).

However, the fraction of inspired oxygen ($F_{IO_{2}}$) used in a normobaric setting to simulate a given altitude is dependent on the accuracy of the calculation of equivalent altitude as a function of the ambient temperature or the model used (Richalet, 2020). Within this framework, the hypoxic challenge test (HCT) is commonly used to evaluate possible development of hypoxaemia associated with air travel, thereby determining whether patients with known lung conditions require oxygen whilst flying (Vohra & Klocke, 1993), owing to the reduced cabin pressure on aeroplanes. Sometimes it is called a fitness‐to‐fly test. European and North American regulatory authorities set the maximum allowable cabin pressure altitude at 2438 m, in order to maintain arterial oxygen saturation at >90% in the average healthy individual; therefore, the British Thoracic Society recommends conducting an HCT by using an inspired gas mixture containing 15% oxygen, which gives an approximately comparable inspired oxygen tension to breathing air at 2438 m; consequently, in‐flight oxygen is recommended if the arterial partial pressure of oxygen ($P_{aO_{2}}$) falls below 6.6 kPa (50 mmHg) or peripheral oxygen saturation ($S_{pO_{2}}$) below 85% within a 20 min test (Coker et al., 2022).

The HCT rationale has been widely used in clinics and research; for example, for the ‘Hypoxia Altitude Simulation Test’ protocol (Dine & Kreider, 2008), the $F_{IO_{2}}$ was set as ∼0.151. In addition to HCT, other types of research have used normobaric hypoxia to simulate what is usually considered very high altitude (≥3500 m a.s.l.,), by setting the $F_{IO_{2}}$ ≤ 0.136, for example, in the study by Saugy et al. (2016). A notorious hypoxia sensitivity test uses a $F_{IO_{2}}$ of 0.115 to quantify the cardiorespiratory response to hypoxia and exercise (Rathat et al., 1992; Richalet et al., 2012). However, are you sure about the altitude you are simulating and the response you are monitoring? This work is aimed to help researchers, clinicians and practitioners working with normobaric hypoxia by arguing the physical aspects of normobaric hypoxia and oxygen concentration, highlighting the underestimated topic of carbon dioxide concentration in confined environments, and raising concerns about the use of single measurements whilst bodily variables fluctuate.

## 2 OXYGEN ESTIMATIONS

Let us hypothetically set one hypoxic condition with $F_{IO_{2}}$ ≈ 0.151 and another with $F_{IO_{2}}$ ≤ 0.136, which ensures high‐altitude conditions whilst allowing all participants to be able to stay in a hypothetical tent for the entire duration of the trial without significant health problems. Beyond the use of a hypoxic chamber with digital control of environmental variables, these hypoxic degrees can be maintained manually by continuously adjusting the hypoxic generators according to the data acquired with an oximeter. During some trials with an altitude generator and an associated sleeping tent (Everest Summit II, Hypoxico, USA), we managed to maintain $F_{IO_{2}}$ at ∼0.151 and $F_{IO_{2}}$ at ∼0.136 thus obtaining on average a $F_{IO_{2}}$ of ∼0.150 and ∼0.134, respectively; in the normoxic condition a mean $F_{IO_{2}}$ of ∼0.203, rather than 0.209, was observed (Gatti et al., 2024). By using the K5 Wearable Metabolic System (COSMED srl, Roma, Italy), we observed a mean temperature (*T*) of ∼28°C, a mean relative humidity (RH) of ∼50% and a mean barometric pressure of ∼755 mmHg. This pressure is plausible, given that our laboratory is at ∼50 m a.s.l. By inserting these data into the online tool (available at https://www.omnicalculator.com/physics/air‐density), a dry air pressure of 740.8 mmHg was calculated. Considering the molar fraction of oxygen mentioned above (0.203, 0.150, and 0.134), the resulting partial pressure of oxygen ($P_{O_{2}}$) was 150.38, 111.12 and 99.27 mmHg, respectively. By using the online tool (available at https://www.omnicalculator.com/physics/air‐pressure‐at‐altitude), which relies on the barometric formula:

(1) $$P=P_{0}\times e^{\frac{-gM\left(h-h_{0}\right)}{RT}}$$

where *M* is the molar mass of dry air (∼2.90 × 10^−2^ kg mol^−1^), *R* is the universal gas costant (8.31432 N m mol^−1^ K^−1^), *h* is the altitude, and *h*_0_ is the sea‐level reference altitude, the moist air pressure at a certain altitude can be estimated, resulting in 572.33 mmHg at 2500 m and 510.95 mmHg at 3500 m. By using the first online tool mentioned above, with *T* = 28°C and RH = 50% the dry air pressure is 558.20 mmHg at 2500 m and 496.80 mmHg at 3500 m, respectively. With the oxygen molar fraction of ∼0.209, we should expect a $P_{O_{2}}$ of 116.92 mmHg at 2500 m and 104.06 mmHg at 3500 m. The above‐mentioned calculations rely on common formulas used to compute air pressure. However, the barometric formula *e*(1) assumes constant temperature; let us use the International Civil Aviation Organization (ICAO) Standard Atmosphere model, which assumes linear temperature change and constant molar mass and gravitational acceleration. The atmospheric temperature lapse, at least in the tropospheric range of the highest Earth peaks, is 6.5°C per 1000 m (this is known as the standard temperature lapse rate, often termed *L*), starting from 15°C at sea level; one can easily use the online calculator (available at https://www.sensorsone.com/icao‐standard‐atmosphere‐altitude‐pressure‐calculator/). By using it, one can assume dry air pressures of 560.17 mmHg at 2500 m and 493.27 mmHg at 3500 m. With the normal oxygen molar fraction, we should expect $P_{O_{2}}$ of 117.33 and 103.32 mmHg, respectively.

In both cases, the values previously computed (111.12 and 99.27 mmHg, respectively) were lower. Therefore, the conditions we used were slightly more hypoxic (lower $P_{O_{2}}$) than a virtual clinician might suppose to reproduce whilst conducting the hypoxic challenge test, and that a virtual trainer might suppose to reproduce whilst conducting a tough hypoxic training session. In particular, after some calculations, the two hypoxic conditions we considered can be comparable to ∼2900 and ∼3900 m a.s.l. with the same experimental conditions of *T* = 28°C and RH = 50%, which are non‐negligible differences!

The partial pressure of inspired oxygen ($P_{IO_{2}}$) refers to the air that interacts with the body at the end of the airways, where temperature and relative humidity are constant (∼37°C and 100%, respectively). By using the ICAO model, we obtain a barometric pressure of 755.51 mmHg at 50 m, then we assume a water vapour pressure of 47.08 mmHg and a dry air pressure of 708.43 mmHg within the airways, hence a $P_{IO_{2}}$ of 106.26 and 94.93 mmHg with $F_{IO_{2}}$ of 0.150 and 0.134, respectively. Recalling the barometric pressures of 560.17 mmHg at 2500 m and 493.27 mmHg at 3500 m from the ICAO model, then considering the water vapour pressure remains at 47.08 mmHg with 37°C and a relative humidity of 100% within the airways, we obtain $P_{IO_{2}}$ of 107.47 at 2500 m and 93.46 mmHg at 3500 m. This is what we can expect by staying outdoors at standard atmosphere during an exposure to altitude (the atmospheric temperature lapse results in a temperature of −1.25°C and −7.75°C at 2500 and 3500 m, respectively). Therefore, the setting recommended by some hypoxic tent/chamber producers might be adequate for simulating altitude hypoxia within the airways of healthy humans. However, the value of 47 mmHg should not be considered as a standard for other animals (King & Agre, 2001) or for patients with an altered structure of the respiratory tract (Primiano et al., 1988).

## 3 CARBON DIOXIDE MATTERS

**FIGURE 1:**
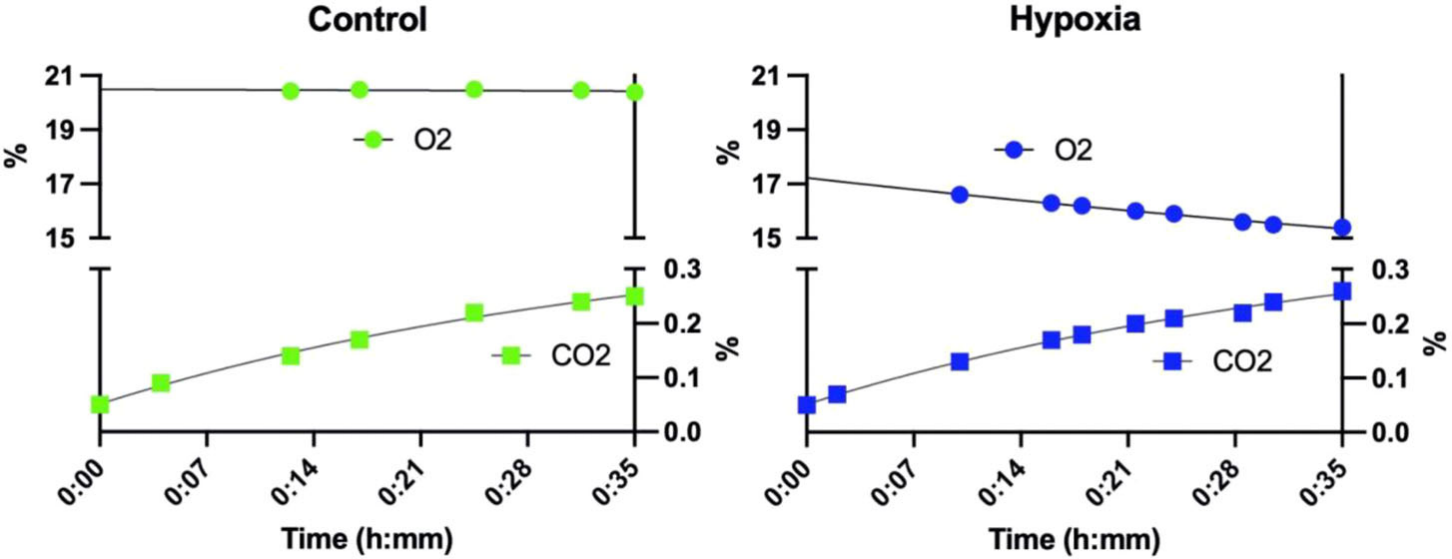
Oxygen and carbon dioxide fraction trajectories during 35 min in conditions of normoxia and normobaric hypoxia.

Another under‐considered aspect relies on CO_2_, which increases during altitude simulation procedures inside tents. To account for this aspect, preliminarily, we measured CO_2_ by using the K5 sensor with one participant inside the tent, comparing one normoxic session with one hypoxic condition. The results are shown in Figure 1. The increase of CO_2_ in the ambient air during the time spent in the tent was non‐linear and followed a one‐phase exponential decay, very similar between the control trial (O_2_ = 20.38%, CO_2_ = 0.25%) and the hypoxic trial (O_2_ = 15.40%, CO_2_ = 0.26%).

The fact that the CO_2_ level did not change can be interpreted speculatively as comparable bioenergetic dynamics during the normoxic trial versus hypoxic trial, but this is outside the scope of this article; here, I focus on the ∼6‐fold increase in CO_2_ levels. It is known that increasing levels of inspired CO_2_ result in non‐linear increases in the partial pressure of arterial CO_2_ and ventilation (Ellingsen et al., 1987), although typically some bodily responses, such as respiratory stimulation and blood pressure increase, are not visible until CO_2_ levels exceed 1%. It is worth mentioning that CO_2_ and O_2_ interactions during Bohr and Haldane phenomena have profound effects on gas exchange that are still subject to interest (e.g., Malte & Lykkeboe, 2018). Therefore, even changes in CO_2_ levels that are not clinically significant have important physiological implications.

Let us assume that we are at sea level (760 mmHg); to simplify the condition, let us assume that we are breathing dry air to eliminate the inter‐individual variations in sweating that would slightly reduce the density of the air as water vapour accumulates inside the tent. At sea level, with a temperature of 20°C, dry air has a density of ∼1.204 kg m^−3^. Carbon dioxide has a density that is ∼1.519 times higher than dry air, i.e., 1.829 kg m^−3^ in our virtual experiment. Let us consider a cubic altitude chamber with a volume of 2 × 2 × 2 = 8 m^3^, which is greater than the volumes of the usual altitude tents. By assuming a fractional inspired CO_2_ of 0.04%, we have 0.32 × 10^−2^ m^3^ of CO_2_, with a resulting mass of 1.829 × 3.2 × 10^−3^ = 5.85 × 10^−3^ kg (5.85 g). The exhaled air has a *T* of 37°C and RH of 100%, thereby resulting in dry air pressure of ∼1.111 kg m^−3^ and CO_2_ pressure of ∼1.688 kg m^−3^. If we assume a normal breath with a tidal volume of 0.5 L (0.5 × 10^−3^ m^3^) and fractional expired CO_2_ of 0.04, the resulting volume of CO_2_ in each exhalation is 2 × 10^−5^ m^3^, with a resulting mass of 1.688 × 2 × 10^−5^ = 3.376 × 10^−5^ kg (3.376 × 10^−2^ g). By assuming the fractional inspired CO_2_ of 0.04 × 10^−2^ (i.e., 100 times lower than fractional expired CO_2_), the CO_2_ mass in each inhalation is 3.376 × 10^−4^ g. Therefore, in each respiratory cycle, the net exhaled CO_2_ mass is 3.376 × 10^−2^ to 3.376 × 10^−4^ = 3.342 × 10^−2^ g. By considering a duration of 1 h and a breathing frequency of 15 breaths min^−1^, one person inside the chamber would exhale 900 times, resulting in a total CO_2_ emission of ∼30.08 g. The resulting volume, given the density we used above, is in the range of 1.645 × 10^−2^ to 1.782 × 10^−2^ m^3^ (with final air in the range of 20°C–37°C), which sums to the initial volume of 0.32 × 10^−2^ m^3^, for a total of 1.965 × 10^−2^ to 2.102 × 10^−2^ m^3^. The percentage of CO_2_ at the end of a 1‐h‐long experiment inside our virtual 8 m^3^ tent would therefore be ∼0.246%–0.263%, which is not a clinically relevant percentage, but non‐negligible for fine‐tuned bodily responses of interest! To simplify the calculation, we can refer to the resting values of exhaled CO_2_ at standard pressure and temperature (sea level and 0°C), i.e., 250 mL min^−1^, then corrected for *T* = 20°C, resulting in 268 mL min^−1^. The increase of CO_2_ accumulation as temperature increases in confined environments owing to a higher exhalation rate has been demonstrated experimentally (Angelova et al., 2021). After 1 h, 16080 mL of CO_2_ would be exhaled, thus 1.608 × 10^−2^ m^3^, similar to the previous estimated value of 1.645 × 10^−2^ m^3^ at 20°C.

If we had used our tent (∼5 m^3^) for this virtual experiment, with the duration we used for CO_2_ measures (35 min) and the ventilation we previously hypothesized (tidal volume = 0.5 L, respiratory frequency = 15 breaths min^−1^), we would have obtained an exhaled CO_2_ mass of 17.55 g (17.55 × 10^−3^ kg). This sums to the initial mass of CO_2_ of ∼3.54 × 10^−3^ kg (given a fractional inspired CO_2_ of 0.04 × 10^−2^ inside a 5 m^3^ tent at 28°C, resulting in a dry air density of 1.165 kg m^−3^), thus resulting in a total CO_2_ mass of ∼21.09 × 10^−3^ kg, i.e., a CO_2_ volume of 0.012 m^3^, which corresponds to a CO_2_ percentage of ∼0.24% in a 5 m^3^ tent. Even considering the adjustments and the individual bioenergetics of the subject inside the tent, the above estimated percentage is highly similar to the ones we measured. It is worth mentioning that an altitude generator, such as the one mentioned above, blows air inwards at a certain rate, in our case ∼100 L min^−1^ (0.1 m^3^), and the outward movement of air is passive, through valves and inlets/outlets. By assuming a constant generation rate of CO_2_ and a uniform mix of air within the confined environment, we can rely on the following simplified mass balance equation for acceptable air quality:

(2) $$Q=\frac{G}{C_{t}-C_{b}}$$

where *Q* isthe ventilation rate, *G* the CO_2_ generation rate, *C*_t_ the target concentration and *C*_b_ the basal concentration of CO_2_. This equation has also been mentioned by the American Society of Heating, Refrigerating and Air‐Conditioning Engineers (ASHRAE) 2001 Standard [as Vo = N/(Cs − Co)]. In our simulation, 11.9 L of CO_2_ have been exhaled in 35 min, thus *G* = 0.34 L min^−1^. Given the initial and final concentrations of CO_2_ (0.04% and 0.25%, respectively), formula e(2) results in 162 L min^−1^ of air being exchanged to maintain CO_2_ at a steady state of 0.25%.

What about night studies? If one considers the possible use of hypoxic chambers/tents (sleeping studies), we can estimate the CO_2_ levels as follows: two participants, duration of sleep 7 h; the estimated exhaled rate of CO_2_ at 20°C (268 mL min^−1^) is supposed to be reduced by 10% during night rest, resulting in ∼241 mL min^−1^; given these conditions, we can expect a total exhaled CO_2_ volume of ∼0.202 m^3^. By assuming a production of ∼3.34 × 10^−2^ g in each breath (tidal volume of 0.5 L), for a breathing rate of 15 breaths min^−1^, the resulting 12 600 exhalations during 7 h would accumulate ∼0.230 m^3^ of CO_2_ at 20°C. If we were to use the hypothetical hypoxic chamber we previously stated (volume of 8 m^3^), it would mean 2.53% + 0.04% = 2.57% in the first estimation and 2.92% CO_2_ in the second estimation inside the tent. According to some statements, the permissible exposure limit for CO_2_ exposure is 0.5% and threshold limit value–short term (i.e., a total weighted average exposure that should not be exceeded at any time during a day) for a 10 min period is 3% (https://www.fsis.usda.gov/sites/default/files/media_file/2020‐08/Carbon‐Dioxide.pdf). By considering that the space is limited and assuming that air is homogeneously mixed within the tent, we can use the simplified equation e(2) as follows: to maintain the permissible exposure limit within 0.5%, assuming a CO_2_ production of 0.68 L min^−1^, an air exchange of ∼147.8 L min^−1^ would be required. It would be better to keep our virtual tent slightly open during our virtual sleep study!

## 4 VOLUMETRIC FRACTION, MOLAR FRACTION AND PARTS PER MILLION

The permissible limit of exposure usually refers to the parts per million (ppm). Given that molar fraction and volumetric fraction are considered to be approximately equivalent in ambient air, the parts per million volume (ppmv) of O_2_ and CO_2_ are simply 210 000 and 400 ppm (21% and 0.04% of 1 million, respectively). Therefore, the CO_2_ permissible limits mentioned above can be expressed as permissible exposure limit < 5000 ppm and threshold limit value–short term < 30 000 ppm. As detailed in Box 1, instead of using the common formula used to convert ppmv in mass per volume units, which assumes dry air at 1 atm and 25°C, one can use the general formula to calculate the concentration of O_2_ and CO_2_ in any conditions: $\mathrm{Concentration}\;\left(\mathrm{mg}\cdot m^{-3}\right)\approx \mathrm{ppmv}\times M_{m}\times P\left(\mathrm{kPa}\right)/8314\cdot T\;(K)$.

**Box 1: In‐depth of the general formula for converting parts per million volumes in mass per volume units and vice versa**

For calculating parts of millions weight (ppmw) from the general gas equation, we initially calculate density (ρ) as follows:

$e\left(3\right)\;\rho \;\left(\mathrm{kg}\;m^{3}\right)=M_{m}\;\left(\mathrm{kg}\;\mathrm{mo}l^{-1}\right)\times P\;\left(\mathrm{Pa}\right)\times R^{-1}\times T^{-1}\;\left(K\right)$

Given *R* (∼8.314 J mol^−1^ K^−1^), at 1 atm (101325 Pa) and 25°C (298.15 K), ρ of CO_2_ (*M*_m_ = 0.04401 kg/mol) is ∼1.799 kg m^−3^ and ρ of O_2_ (*M*_m_ = 0.032 kg mol^−1^) is ∼1.308 kg m^−3^. Given that ρ=m/V, 1 m^3^ of dry air with volumetric fractions of 21% of O_2_ and 0.04% of CO_2_ results in 0.27468 kg (274 680 mg) of O_2_ and 0.0007196 kg (719.6 mg) of CO_2_. The total mass of 1 m^3^ of dry air (*M*_m_ = 0.02896 kg mol^−1^) at 1 atm and 25°C (i.e., ρ ≈ 1.184 kg m^−3^) is 1.184 kg (1 184 000 mg). Therefore, simple proportions for calculating ppmw reveal ∼231 993 ppmw of O_2_ and ∼608 ppmw of CO_2_.

The following formula is commonly used to convert ppmv (as a surrogate of molar ppm) in mass per volume units as:

$e\left(4\right)\;\mathrm{Concentration}\;\left(\mathrm{mg}\;m^{-3}\right)=\frac{\mathrm{Concentration}\;\left(\mathrm{ppm}\right)\times \;\mathrm{molar}\;\mathrm{mass}\;\left(g\;\mathrm{mo}l^{-1}\right)}{24.45\left(L\;\mathrm{mo}l^{-1}\right)}$

The constant of 24.45 refers to the molar volume (*V*_m_) of dry air at 1 atm and 25°C, which can be calculated as:

$e\left(5\right)\;V_{m}\left(L\;\mathrm{mo}l^{-1}\right)=M_{m}\;\left(\mathrm{kg}\;\mathrm{mo}l^{-1}\right)\times 1000/\rho \left(\mathrm{kg}\;m^{-3}\right)$

Of course, if barometric pressure and temperature are different, one can use e(3) and e(5) to calculate ρ and *V*_m_ of dry air, respectively. Indeed, by combining e(3), e(4) and e(5) and leaving *M*_m_ in grams per mole as conventionally reported, we can simplify the conversion of ppmv to milligrams per metre cubed in dry air with any conditions of pressure and temperature, thus proposing the following equations:

$e\left(6\right)\;\mathrm{Concentration}\;\left(\mathrm{mg}\;m^{-3}\right)\;∼\frac{\mathrm{ppmv}\;\times \;M_{m}\times P\;\left(\mathrm{Pa}\right)}{8314\;\times \;T\;\left(K\right)}$

And consequently

$e\left(7\right)\;\;\mathrm{pp}m_{\left(X\right)}∼\frac{\left[X\right]\;\left(\mathrm{mg}\;m^{-3}\right)\times \;8314\times T\left(K\right)}{M_{m\left(x\right)}\;\times \;P\;\left(\mathrm{Pa}\right)}$

To give some examples, we consider, as usual, 21% O_2_ (210 000 ppm) and 0.04% CO_2_ (400 ppm):

- Example 1 (normoxia, altitude of 100 m a.s.l.): *T* = 25°C (298.15 K), barometric pressure = 751.341 mmHg, RH = 50%, dry air pressure = 739.5 mmHg (98 592 Pa), we will find the air filled with 267 280 mg m^−3^ of O_2_ and 700 mg m^−3^ of CO_2_.
- Example 2 (hypoxia, altitude of 3500 m a.s.l.): *T* = 0°C (273.15 K), barometric pressure = 490.57 mmHg, RH = 35%, dry air pressure = 489 mmHg (65 195 Pa), we will find the air filled with 192 918 mg m^−3^ of O_2_ and 505 mg m^−3^ of CO_2_.

## 5 FLUCTUATIONS MATTER

When individuals are at altitude, self‐adjustments give rise to fluctuations in physiological variables.

Responses and fluctuations of diverse physiological variables vary in magnitude and time courses, and this greatly affects the interpretation of data; as an example, the typical response of increased heart rate can be observed at lower simulated altitude in the case of rising altitude than at a steady altitude; another example relies on different time delay when comparing the respiratory response, which is faster at lower altitudes, with the oxygen saturation response, which exhibits a faster response at higher altitudes (Zhang et al., 2024).

Variability per se has been considered as a proxy of the physiological response to hypoxia. For example, it has been reported how induced oscillations in arterial blood pressure preserve cerebral tissue oxygen saturation during sustained hypoxia (Anderson et al., 2021). All physiological signals show fluctuations, but oxyhaemoglobin saturation dynamics are particularly of interest within the topic of hypoxia. Bhogal and Mani (2017) reported that higher $S_{pO_{2}}$ is associated with a more regular pattern of variability and lower complexity and that the amount of overall variability varies across individuals. Costello et al. (2020) suggest moving beyond linear measures of dispersion, such as standard deviation, thus computing other indices, such as sample entropy (which quantifies the degree of irregularity), detrended fluctuation analysis (which quantifies the self‐similarity) and multiscale entropy (which extends sample entropy analysis to multiple time scales), because fluctuations in physiological time series are usually not linear. This approach has also been followed recently by Montull et al. (2025), who demonstrated that the randomness of the muscle oxygenation signal increases whilst approaching physical exhaustion.

**FIGURE 2:**
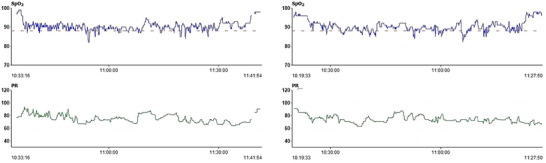
Peripheral oxygen saturation ($S_{pO_{2}}$) and photoplethysmogram‐derived pulse rate (PR) of one healthy young adult during a 1 h exposure to normobaric hypoxia (fraction of inspired O_2_ ≈ 0.133–0.138).

The sigmoidal shape of oxyhaemoglobin saturation, mostly attributable to a co‐operative reversible phenomenon, has attracted the interest of physicists and mathematicians. Several models have been proposed to define the relationship that describes oxygen binding by haemoglobin, such as the prototypical Hill empirical equation and subsequent models (Lavrinenko et al., 2023). From the sigmoidal shape, it is evident that any perturbation of $P_{O_{2}}$ (*x*‐axis) in the rapidly ascending/descending portion of the curve would result in a greater change of oxyhaemoglobin saturation (*y*‐axis) than in those portions of the curve approaching the steady state, as for values of $S_{pO_{2}}$ of >95%. Although exposed to hypoxia, values are thus plausibly more susceptible to variability, and the use of spot values should be discouraged. To determine how a spot value can strongly bias our interpretation, Figure 2 shows $S_{pO_{2}}$ and heart rate signals obtained from the finger pulse oximeter Beurer PO80 (Beurer GmbH, Ulm, Germany) on one healthy young participant exposed twice to normobaric hypoxia ($F_{IO_{2}}$ ≈ 0.133–0.138), whilst remaining in a resting state. The use of averages over longer periods is a superior approach in comparison to the use of spot values. Nevertheless, this constitutes a limitation, because it fails to represent the dynamics of the system.

Given that $S_{pO_{2}}$ is largely used as a prompt index of hypoxia‐induced effects, common pitfalls that might bias the validity should be considered seriously (i.e., greater inaccuracy as values decrease, skin pigmentation, perfusion, body movements, rate and rhythm of breathing, and operating temperature; Prosperi et al., 2023). A worth mentioning argument relies on the type of haemoglobin, because both glycosylated haemoglobin (HbA1c) and carboxyhaemoglobin (HbCO) exhibit a higher affinity for oxygen, thus determining a leftward shift of the curve (Marschner & Rietbrock, 1994, 1995). Therefore, although the effects of elevated levels of HbA1c and HbCO on the dynamics of oxyhaemoglobin dissociation might be counterbalanced by other factors, it would be appropriate always to report the presence of participants such as diabetics and smokers.

## 6 LIMITATIONS AND PERSPECTIVES

Regarding the aim of this article, molar fraction and volumetric fraction are considered to be approximately equivalent in air. Oxygen and nitrogen usually behave as ideal gases when the temperature is not extremely low and the pressure is not extremely high. However, carbon dioxide behaves a bit differently; if this behaviour can be considered negligible in ambient air, the increased levels of carbon dioxide can impact the macroscopic features of air, and the volumetric fraction should be lower than the molar fraction. The simplified mass balance equation e(2) assumes a constant generation rate of substance (here CO_2_) and a perfect mixing of air, thereby being independent from the volume of the space; this can be assumed for resting individuals in a highly confined environment, but for more complex conditions, models of computational fluid dynamics should be used.

Concerning the hypoxic challenge test, Billings et al. (2013) reported a set of linear and non‐linear equations, the latter being more reliable, which can determine whether a patient should receive supplemental oxygen, without the need for an HCT; the relative contributions of predictivity were reported to be greater for $P_{aO_{2}}$, followed by the partial pressure of arterial CO_2_ and weight. The use of these equations might extend and overcome some pitfalls of the HCT. Moreover, predictive equations might be matched to the results of HCT to verify the putative alignment, thereby obtaining additional insights into the individual response to hypoxia.

## 7 CONCLUSION

In the debate about normobaric versus hypobaric hypoxia, it has been argued that the duration of exposure, type of task, method of calculating the hypoxic dose, and issue of confinement all influence the comparisons between normobaric hypoxia and hypobaric hypoxia (Girard et al., 2012). To ascertain any differential bodily effects, Richalet recommended matching the same $P_{aO_{2}}$ (Richalet, 2020), whereas Millet and Debevec (2020) matched the same $P_{IO_{2}}$. Despite the divergences, there is consensus in describing in detail the methodological approach and estimates used, in addition to reporting all relevant environmental variables, such as altitude, barometric pressure, relative humidity and temperature.

To ensure that the normobaric setting reliably represents a simulated altitude, the values for temperature, humidity and barometric pressure should always be considered and reported. Whether considering the match for $P_{IO_{2}}$ or $P_{aO_{2}}$, the correct estimation for simulating altitude assumes that the airway mucosa is healthy. In fact, humidification of the airways to maximum RH requires that the mucosa be sufficiently hydrated, which is not obvious in acute or chronic pathological conditions. The other assumption is that the internal temperature is 37°C, which instead might vary slightly between individuals and might decrease in simulated conditions where a cold ambient temperature is reproduced. All these considerations require the environmental conditions and the state of health of the participants’ airways to be reported correctly.

The ppm of CO_2_ in ambient air should be checked before any experiments if possible, and exhaled CO_2_ should be controlled or estimated; values should be maintained below the permissible exposure limits. In addition, during hypoxic states, even small variations in CO_2_ can affect arterial oxygen saturation much more markedly than in normoxic conditions, because the oxyhaemoglobin dissociation curve is in its steep portion. For this reason, it is advisable that CO_2_ levels are always monitored and reported.

Finally, regarding fluctuations, it is worth mentioning that the stress response and constant remodelling define bodily systems in a homeodynamic, rather than homeostatic, space (Rattan, 2020), whose capacity might alter the acute response to hypoxia, as in ageing (Bevere et al., 2022). If only spot values are considered, on the one hand, there is a risk of misjudgement for highly variable values, such as arterial oxygen saturation in hypoxia, and on the other hand, the oscillatory characteristics of a homeodynamic system are masked, which is an issue that also affects averaged values over long‐lasting measurements.

To answer the question, ‘Are you sure about the altitude you are simulating and the response you are monitoring?’, one should: (1) consider that usually a normobaric hypoxia setting underestimates the environmental hypoxia of a target simulated altitude; (2) calculate the amount of CO_2_ in the inspired air as duration increases, or estimate it by using the proposed methods and formulas; and (3) avoid the use of spot measurements or averaged values, particularly those of $S_{pO_{2}}$, because fluctuations matter greatly. By using the considerations and suggestions of this article, the use of hypoxic tests to evaluate the physiological response will be more valid and informative, or at least let us assume it.

## AUTHOR CONTRIBUTIONS

Conceptualization, methodology, formal analysis, writing ‐ original draft, visualization: Danilo Bondi.

## CONFLICT OF INTEREST

The author declares no conflicts of interest.

## FUNDING INFORMATION

The author declares no specific funding for this work.

## Data Availability

For this article, only illustrative data related to other projects or assumptions for the calculations were used.
